# Loss of Function of Scavenger Receptor SCAV-5 Protects *C. elegans* Against Pathogenic Bacteria

**DOI:** 10.3389/fcimb.2021.593745

**Published:** 2021-08-03

**Authors:** Aixiao Luo, Huiru Jing, Lei Yuan, Yanzhe Wang, Hui Xiao, Qian Zheng

**Affiliations:** Key Laboratory of the Ministry of Education for Medicinal Plant Resources and Natural Pharmaceutical Chemistry, National Engineering Laboratory for Resource Development of Endangered Crude Drugs in the Northwest of China, College of Life Sciences, Shaanxi Normal University, Xi’an, China

**Keywords:** scavenger receptors, SCAV-5, *C. elegans*, innate immunity, pathogenic bacteria

## Abstract

Scavenger receptors play a critical role in innate immunity by acting as the pattern-recognition receptors. There are six class B scavenger receptors homologs in *C. elegans.* However, it remains unclear whether they are required for host defense against bacterial pathogens. Here, we show that, of the six SCAV proteins, only loss of function *scav-5* protect *C. elegans* against pathogenic bacteria *S. typhimurium* SL1344 and *P. aeruginosa* PA14 by different mechanism. *scav-5* mutants are resistant to *S. typhimurium* SL1344 due to dietary restriction. While *scav-5* acts upstream of or in parallel to *tir-1* in conserved PMK-1 p38 MAPK pathway to upregulate the innate immune response to defend worms against *P. aeruginosa* PA14. This is the first demonstration of a role for SCAV-5 in host defense against pathogenic bacteria. Our results provide an important basis for further elucidating the underlying molecular mechanism by which *scav-5* regulates innate immune responses.

## Introduction

Scavenger receptors are eight classes transmembrane glycoproteins that first defined by its ability to bind and subsequently internalize modified low-density lipoproteins (mLDL) (PrabhuDas et al., 2017). Later, a large repertoire of ligands such as lipoproteins, cholesterol esters, phospholipids, proteoglycans, and carbohydrates have been identified that can be recognized by Scavenger receptors. Thus, Scavenger receptors are involved in an impressively broad range of functions including lipid metabolism, antigen presentation, phagocytosis, clearance of apoptotic cells, and innate immunity (Silverstein and Febbraio, 2009; Graham et al., 2011; He et al., 2011; Canton et al., 2013). Innate immunity is the first line of defense against pathogens, which is critical to maintain homeostasis, prevent infection, and activate the adaptive immune response. The components of innate immunity include external physical and chemical barriers, mucous membranes, internal humoral and, cellular effector mechanisms (Riera Romo et al., 2016). It is now clear that many scavenger receptors can recognize conserved patterns unique to microbial surfaces that are referred to as pathogen-associated patterns (PAMPs) (Mukhopadhyay and Gordon, 2004; Pluddemann et al., 2006; Pluddemann et al., 2011). On the other hand, scavenger receptors can be used as co-receptors by bacteria and viruses for entry into host cells (Haraga et al., 2008; Hawkes et al., 2010; Que et al., 2013).

The nematode *Caenorhabditis elegans* has been used as a model for studying bacterial virulence and innate immunity (Irazoqui et al., 2010). Despite lacking both Toll and Imd pathways as well as the adaptive immune system (Anderson et al., 1985; Gottar et al., 2002), *C. elegans* has developed special mechanisms that include innate immune response, bacteria avoidance behavior, and RNA interference to defend against pathogenic bacteria throughout evolution (Aliyari and Ding, 2009; Taffoni and Pujol, 2015). In fact, the absence of adaptive immune response makes *C. elegans* very useful for dissecting the innate immune mechanisms in pathogen-host interactions. The primary food source of *C. elegans* in laboratory is the *E. coli* strain OP50, but other bacteria and fungi also support its growth and reproduction. Several feed-based infection models have been established, such as *Salmonella typhimurium* and *Pseudomonas aeruginosa (* Tan et al., 1999; Aballay et al., 2000), to study the regulation between intestinal infection and innate immunity (Alegado et al., 2011; Kirienko et al., 2013; Curt et al., 2014). *Salmonella typhimurium* SL1344 proliferates and establishes a persistent infection in the intestine of *C. elegans*, resulting in the eventual death of the worm by actively inhibiting innate immune pathways (Aballay et al., 2000; Desai et al., 2019). The human pathogen *Pseudomonas aeruginosa* PA14 kills worms within 4–24 hours by the production several diffusible toxins in ‘fast killing’ model (Cezairliyan et al., 2013), ultimately suppresses the FOXO/DAF-16 innate immunity pathway in the intestine of *C. elegans (* Zheng et al., 2021). The innate immunity in *C. elegans* is regulated by several major pathways to defense against pathogens including PMK-1 p38 MAPK pathway. In the PMK-1 pathway, TIR-1 functions upstream of the NSY-1–SEK-1–PMK-1 p38 MAPK cascade (Kim et al., 2002; Couillault et al., 2004; Andrusiak and Jin, 2016). In this cascade, NSY-1 phosphorylates SEK-1, SEK-1 phosphorylates PMK-1, and finally activates PMK-1 to regulate pathogen response genes (Tanaka-Hino et al., 2002).

SCAV-1-6 are the six scavenger receptors homologs in *C. elegans*, belong to class B scavenger receptors (SRB). There are three members in SRB in mammals including CD36, SRB-I/II (SRB-II is a splicing variant of SRB-I), and lysosomal integral membrane protein 2 (Means et al., 2009). SRB has been reported to be involved in innate immunity (Feng et al., 2011). However, whether SCAV-1-6 have effects on defense against pathogenic bacteria in *C. elegans* and the underlying mechanism remains unclear.

Here, we investigate the functions of SCAV-1-6 in innate immunity in *C. elegans*. We find that *scav-5* mutants can effectively defend *C. elegans* against pathogenic bacteria *S. typhimurium* SL1344 and *P. aeruginosa* PA14. In addition, we show that the mechanism of protecting *C. elegans* against the above two pathogenic bacteria is quite different by *scav-5* loss of function. The *scav-5* mutants displayed reduced pharyngeal pumping after *S. typhimurium* SL1344 infection, which lead to dietary restriction and extended lifespan. While, after *P. aeruginosa* PA14 infection, innate immune response was activated through PMK-1 p38 MAPK pathways in *scav-5* mutants. Moreover, our genetic epistatic analysis indicates that *scav-5* may function upstream of, or in parallel to *tir-1* in PMK-1 innate immune response pathway to protect *C. elegans* against *P. aeruginosa* PA14. Our results provide an important basis for further elucidating the underlying mechanism of how *scav-5* regulates innate immune response.

## Materials And Methods

### *C. elegans* and Bacterial Strains

The *C*. *elegans* strains used in this study were N2, *scav-2(ok877)*, *scav-3(ok1286)* and *scav-5(ok1606)*. *C*. *elegans* were maintained on NGM plates with *E*. *coli* OP50 or HT115 bacteria.

### RNAi of *scav-1, scav-4, scav-6*

Three RNAi constructs were created using 700 bp, 899 bp and 730 bp segments of scav-1, scav-4, scav-6 coding region (bases 1 to 700, 3101 to 4000, and 1to 730, respectively), which were amplified by PCR and cloned into the vector pPD129.36, These plasmids were transformed into the RNAi bacteria strain HT115, and RNAi experiments were carried out with these strains following established protocols (Timmons et al., 2001) by using HT115 bacteria expressing the empty vector pPD129.36 as the control. For all RNAi experiments, the synchronization egg of the old-adult N2 spawn on NGM plates with RNAi bacteria, and L4 stage animals were used for subsequent studies, as described in more detail below.

### *C. elegans* Pathogenic Bacteria Infection Assay

*S. typhimurium* SL1344 were pipetted on NGM plates and incubated overnight. *P. aeruginosa* PA14 infect *C.elgans* with slow killing way, which *P. aeruginosa* PA14 were pipetted on 0.35% NGM low osmotic pressure medium. Plates were incubated for 24 hours at 37°C, and 24 hours at 25°C (Shivers et al., 2010). *C.elegans* were cultured on these plates based on eating pathogenic bacteria established infection model.

### Measurement of Lifespan and Heat Resistance

The indicated genotypes old-adult animals spawn on NGM plates with *E*. *coli* OP50, and L4 stage animals were picked on new NGM plates with *E*. *coli* OP50. Each NGM plate culture approximately 25 animals and each group amount have 80 animals at least. Animals of L4 stage as zero-day in lifespan count. Thermotolerance assays were performed as described (McColl et al., 2010). Briefly, WT and *scav-5(ok1606)* L4 stage hermaphrodites were infected with *S. typhimurium* SL1344 and *P. aeruginosa* PA14 for 24 hours respectively, adults were transferred to 35°C and scored as alive or dead based on responding to prodding with a platinum wire after 12 hours.

### Bacterial Avoid Behavior

Small, uniform circular bacterial lawns were prepared by pipetting 100 uL of overnight cultures of *E*. *coli* OP50 or *S. typhimurium* SL1344, *P. aeruginosa* PA14 on the center of NGM dishes and allowing at least one day to dry. To score each animal as inside or outside the lawn (Avoidance=N_out_/N_total_) at larval stages (L1 to L4) and young adults (YA), we transfer 50-80 eggs to the bacterial lawn without disturbing or spreading the lawn and record avoidance of developmental stage.

### Pharyngeal Pump

Pharyngeal pumping was assessed by observing the number of pharyngeal contractions during a 10-sec interval for longitudinal studies or a 60-sec interval. The experimental details of observing the number of pharyngeal contractions were determined as described (Kumar et al., 2016).

### Bacterial Colonization

We transferred plasmid pET28a into DH5α through heat shock and transferred plasmid ptfLC3 into *P. aeruginosa* PA14 through electric shock. L4 stage WT and *scav-5(ok1606)* were cultured on DH5α-GFP or *S. typhimurium* SL1344-GFP, *P. aeruginosa* PA14-GFP continuing 48 h and following observation the bacterial colonization in intestine by microscope.

### Quantitative RT-PCR

To generate synchronous populations of worms for RNA extraction, we bleached WT and *scav-5(ok1606)* adults to collect eggs and cultured eggs at 20°C on NGM dishes spread with *E. coli* OP50. These synchronized worms were washed and collected for RNA isolation. Briefly, RNA was isolated using a Trizol (Invitrogen) and chloroform extraction (reagent Phenol: chloroform: isoamyl alcohol, 25:24:1 was used to chloroform extraction and isopropanol was used to precipitation RNA). RNA was diluted in nuclease-free water and quantified using a NanoDrop. cDNA was synthesized using HiScript II Q RT SuperMix kit (Vazyme). Real-time PCR was performed using an Applied Biosystems Step One Plus Real-Time PCR system and SYBR green master mix. mRNA fold change was calculated using the comparative CT method (Schmittgen and Livak, 2008) by comparing mRNA levels of the internal control gene *tbg-1*.

### Immunoblot Analyses

Harvested worms were put in a glass homogenizer with RIPA buffer for grinding adequately. The lysate was centrifuged and supernatant protein was transferred into the new tube. Protein was quantified using BCA protein assay kit. And the total protein from each sample was electrophoresis on PAGE 10% gels, transferred to nitrocellulose membranes, blocked with 5% powdered milk in TBST, and probed with a 1:1000 dilution of an antibody that recognizes the phosphorylated of PMK-1 (Cell Signaling Technology Corporation). The blot was then stripped and reprobed with a 1:1000 dilution of an anti-actin antibody. Anti-rabbit, and anti-mouse IgG secondary antibodies were used to detect the primary antibodies following the addition of ECL reagents (Thermo Fisher Scientific, Inc.) which were visualized using a luminescence instrument.

### Membrane Yeast Two-Hybrid (MYTH) Assay

*scav-5* cDNA was inserted between the PstI and XbaI site of vector pMetYCgate and tir-1 cDNA was inserted between the EcroI and XmaI site of vector pNubXgate32-HA. The fusion plasmids were transferred into yeast strain AP4 and picked individual yeast colony into medium and incubated overnight, diluted it into 10^-1^, 10^-2^ and 10^-3^ respectively, dropped onto SD-Trp-Leu-His-Ade medium by 10 uL each drop. Images of yeast were taken after culturing at 30°C for 3 days.

### Microscopy

Nematodes were mounted onto 2% agar pads, paralyzed with levamisole and photographed using an AXIO Imager Z1 microscope. Photographs were acquired using the same imaging conditions for a given experiment, and were processed in Photoshop.

### Statistical Analyses

All data from lifespan, heat stress resistance, avoidance, pharyngeal pump, immunoblot and qRT-PCR analyses were repeated by three biological duplications, and were analyzed using the unpaired two-tailed Student’s t-test. “*” means P< 0.05; “**” means P<0.01; “***” means P<0.001, “****” means P<0.0001. Error bars in each column diagram represent SEM.

## Results

### All Members of the Scavenger Receptors Family Except SCAV-3 Are Expressed in the Intestinal Tissue in *C. elegans*

**Figure 1:**
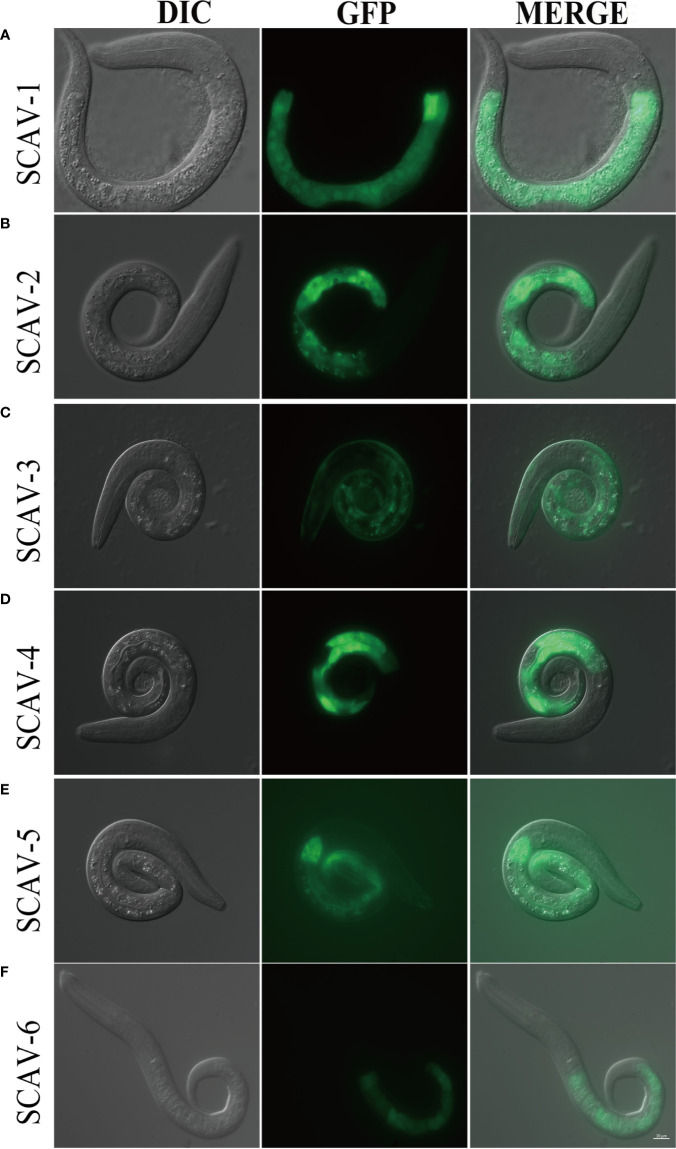
The expression pattern of scavenger receptors in *C.elegans*. **(A–F)**: Tissue profiling of scavenger receptor SCAV-1-6. DIC images of L1 stage live transgenic animals (left). Green fluorescence images show tissue localization of scavenger receptor SCAV-1-6 (middle). Merge images (right). Scale bar=10 μm.

To investigate the role of scavenger receptors family in innate immunity in *C. elegans*, we first set out to define the tissues in which they are expressed. We generated plasmids that express green fluorescent protein (GFP) under the control of around 3 kb proximal promoter of *scav-1-6*, and got exchromosomal transgenic strains by microinjection. We observed strong GFP expression in intestine tissues, which were driven by *scav-1, scav-2, scav-4, scav-5*, and *scav-6* promoter (**Figures 1A, B, D–F**). While SCAV-3 was expressed in all tissues, which is consistent with a previous study (**Figure 1C**) (Li et al., 2016). Intestinal epithelial cells provide an essential line for *C. elegans* against ingested pathogens (Gravato-Nobre and Hodgkin, 2005). Since immune response to pathogenic bacteria was measured from when they reached young adult stage, we further observed *in vivo* expression of scavenger receptors in L4 worms, and found that SCAV-1, SCAV-2, SCAV-4, SCAV-5 and SCAV-6 were expressed in intestine tissues (**Figure S1**). Thus, our expression pattern results indicate that *scav-1, scav-2, scav-4, scav-5*, and *scav-6* may be involved in defense against pathogenic bacteria.

### Loss of Function of scav-5 Protects *C. elegans* Against Pathogenic Bacteria Infection by *P. aeruginosa* PA14 and *S. typhimurium* SL1344

**Figure 2:**
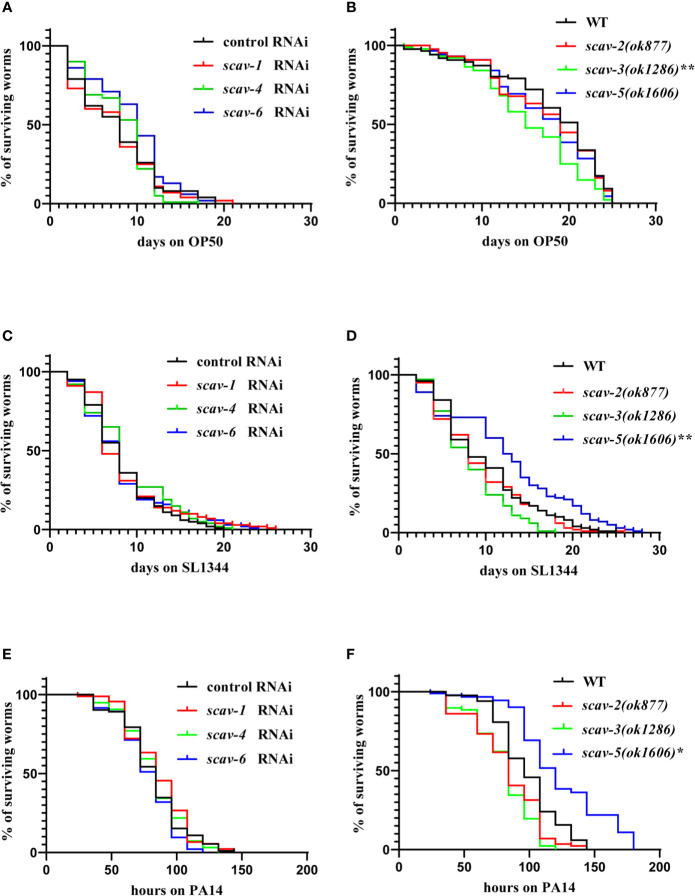
*scav-5* mutants are resistant to *S. typhimurium* SL1344 and *P*. *aeruginosa* PA14. **(A, B)** The lifespan of wild type with *scav-1*, *scav-4*, *scav-6* RNAi **(A)** and *scav-2*, *scav-3*, *scav-5* mutants **(B)** fed on *E. coli* OP50. **(C, D)** The *scav-5(ok1606)* mutation significantly extended the lifespan of otherwise wild type, *scav-1*, *scav-4*, *scav-6* RNAi **(C)** and *scav-2(ok877)*, *scav-3(ok1286)* mutants **(D)** fed on *S. typhimurium* SL1344. (P < 0.01) **(E, F)** The *scav-5(ok1606)* mutation significantly extended the lifespan of otherwise wild type, *scav-1*, *scav-4*, *scav-6* RNAi **(E)** and *scav-2(ok877)*, *scav-3(ok1286)* mutants **(F)** fed on *P. aeruginosa* PA14. (P < 0.05).

To examine the interaction between scavenger receptors and innate immune response in *C. elegans*, we used scavenger receptors mutants *scav-2(ok877)*, *scav-3(ok1286)*, *scav-5(ok1606)* and *scav-1*, *scav-4*, *scav-6* RNAi-treatment wide type (WT) worms, and analyzed their lifespan when feeding on non-pathogenic bacteria OP50. We observed that only the lifespans of *scav-3(ok1286)* mutants were significantly reduced compared to the wild-type N2 (**Figures 2A, B**). Next, we analyzed their lifespan after *P. aeruginosa* PA14 and *S. typhimurium* SL1344 infection. We found that only the lifespans of *scav-5(ok1606)* mutants were significantly extended than control (**Figures 2C–F**). Many *C. elegans* mutations that delay aging also increase stress resistance (Wu et al., 2002; McColl et al., 2005; Saier et al., 2018). To test the effect of *scav-5* mutation on stress resistance, we monitored heat resistance at 35°C of *scav-5* mutants fed on *E. coli* OP50, *S. typhimurium* SL1344 and *P. aeruginosa* PA14 respectively. Our results shown that *scav-5* mutant animals have significantly increased thermotolerance compared to WT when feeding on OP50 and *S*. *typhimurium* SL1344. We did not observe any survival of wide type and *scav-5* mutants by heat stress treatment when feeding on *P. aeruginosa* PA14, which may due to the strong toxicity of PA14 (**Figure S2A**). *scav-5(ok1606)* is likely to be a strong loss of function or null allele based on the lack of detectable mRNA, we used this allele for our further analysis (**Figures S2B, S2C**). To confirm that loss of function of SCAV-5 was responsible for defense against pathogenic bacteria in *scav-5(ok1606)* mutants, we generate transgenic strains *scav-5(ok1606)*;P*_scav-5_*SCAV-5 that expresses SCAV-5 by its promoter by injecting plasmids into *scav-5(ok1606)* mutants, and analyzed their lifespan fed on *S. typhimurium* SL1344 and *P. aeruginosa* PA14 respectively. We found that re-expresses SCAV-5 in *scav-5(ok1606)* mutants abolished its protection effects after *S. typhimurium* SL1344 and *P. aeruginosa* PA14 infection (**Figure S3**). Our results demonstrated that loss of function of SCAV-5 defend *C. elegans* against pathogenic bacteria infection.

### *scav-5* Mutants Are Resistant to *S. typhimurium* SL1344 by Dietary Restriction

**Figure 3:**
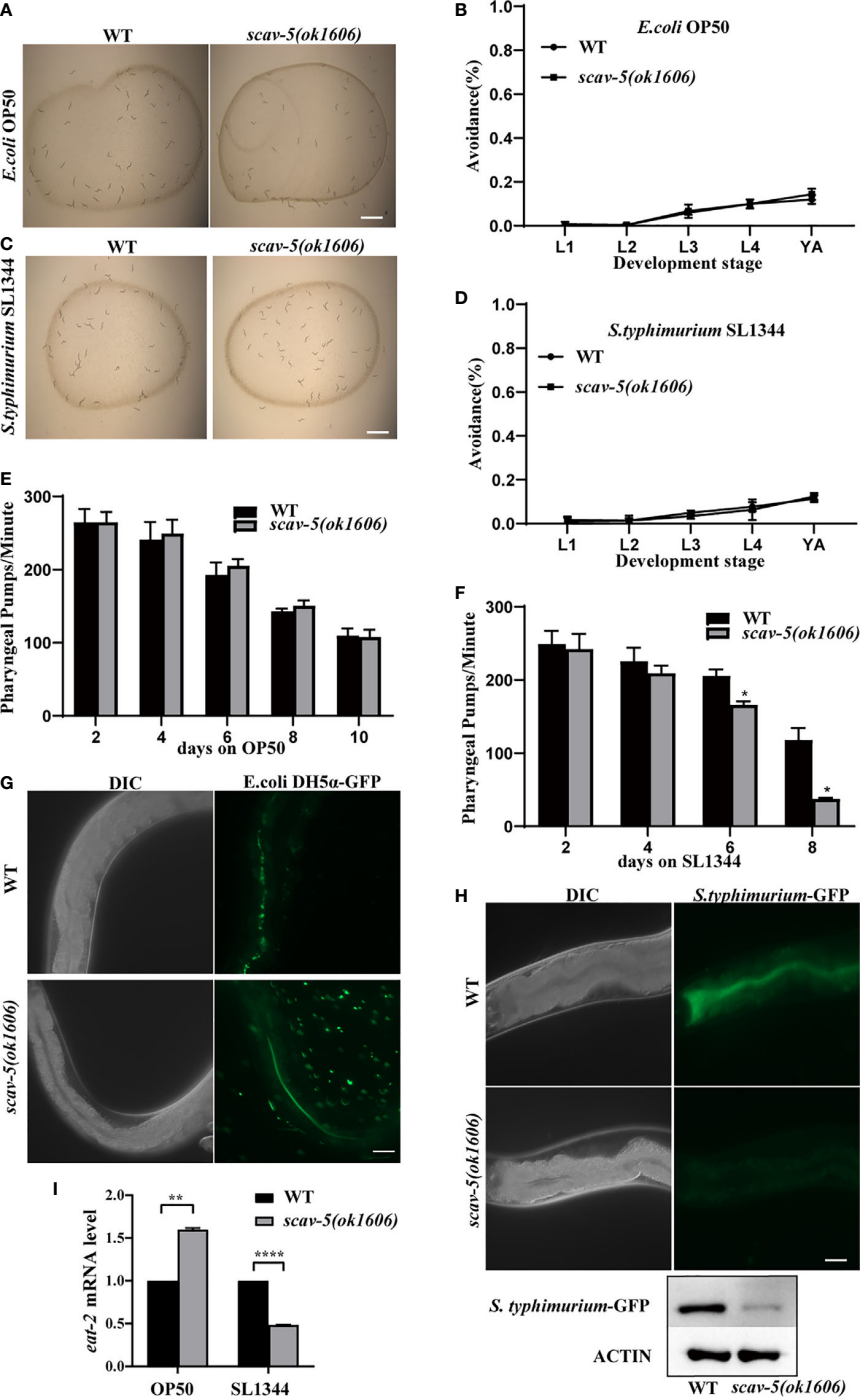
*scav-5* mutants display dietary restriction when cultured on *S. typhimurium* SL1344. **(A, C)** Bright field photographs of wild type and *scav-5(ok1606)* mutant cultured on *E. coli* OP50 **(A)** and *S. typhimurium* SL1344 **(B)** both have no avoidance behavior at L4 stage. Scale bar=100 μm. **(B, D)** Quantification of avoidance rate of *scav-5(ok1606)* mutants cultured on *E. coli* OP50 **(B)** and *S. typhimurium* SL1344 **(D)** from L1 to young adult stage. **(E, F)** Pharyngeal pumps per min of wild type and *scav-5(ok1606)* mutants fed with *E. coli* OP50 **(E)** and *S. typhimurium* SL1344 **(F)**. **(G, H)** The DIC and GFP fluorescence image of partial intestine in wild type and *scav-5(ok1606)* mutant cultured on *E. coli* OP50 **(G)** and *S. typhimurium* SL1344 **(H)** 48 h after L4 stage. Scale bar=20 μm. **(I)** qRT-PCR analysis of transcription level of DR gene *eat-2* in wild-type and *scav-5(ok1606)* animals cultured on *E. coli* OP50 or *S. typhimurium* SL1344.

To determine the mechanism by which *scav-5* mutants are resistant to *S. typhimurium* SL1344, we first quantified their bacterial lawn avoidance behavior. We found that *scav-5* mutants animals did not display bacterial avoidance behavior when cultured on *E. coli* OP50 or *S. typhimurium* SL1344 (**Figures 3A–D**). Next, we analyzed the pharyngeal pumping rate of *scav-5* mutants when fed on *E. coli* OP50 or *S. typhimurium* SL1344 by using a dissecting microscope. We observed that *scav-5* mutants can pump at a rate comparable to wild type when fed on *E. coli* OP50 (**Figure 3E**). While *scav-5* mutants displayed a substantial age-related reduction of pharyngeal pumping rate when cultured on *S. typhimurium* SL1344 (**Figure 3F**). These imply that the reduced pharyngeal pumping rate in *scav-5* mutants when feeding on *S. typhimurium* SL1344 leads to reduced bacterial ingestion, and then results in dietary restriction. To further confirm the dietary restriction in *scav-5* mutants when feeding on *S. typhimurium* SL1344, we detected the *E. coli* DH5α and *S. typhimurium* colonization in the intestine of *scav-5* mutants by feeding with *E. coli* DH5α-GFP and *S. typhimurium*-GFP for 48h after L4 stage respectively. *scav-5* mutants intestine displayed significantly decreased fluorescence intensity when cultured on *S. typhimurium*-GFP (**Figures 3G, H**), the decreased level of S. *typhimurium*-GFP in *scav-5* mutants was confirmed by western blotting (**Figure 3H**). Furthermore, we observed the mRNA level of *eat-2*, which is required for pharyngeal pumping rate and subsequent food intake (McKay et al., 2004), in WT and *scav-5* mutants when feeding on *S. typhimurium* SL1344, and found the transcriptional level of *eat-2* was reduced in *scav-5* (**Figure 3I**), these results demonstrate that *scav-5* mutation protects worms against *S. typhimurium* SL1344 by dietary restriction.

### Loss of Function of scav-5 Does Not Upregulate Defense Genes Expression in *C. elegans* Infected With *S. typhimurium* SL1344

**Figure 4:**
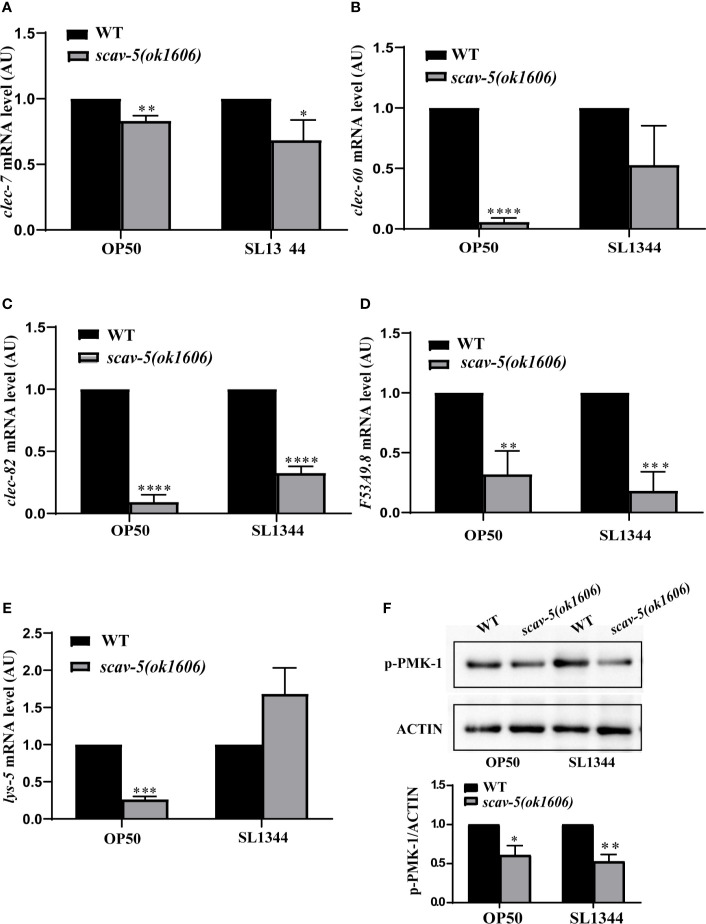
The innate immune response in *scav-5* mutants fed on *E. coli* OP50 or *S. typhimurium* SL1344 were downregulated. **(A)** Immunoblot analysis of p-PMK-1 in young adult animals of wild type and *scav-5(ok1606)* mutants fed on *E. coli* OP50 or *S. typhimurium* SL1344 for 48h. **(B–F)** qRT-PCR analysis of transcription level of pathogenic response genes *clec-7*, *clec-60*, and *clec-82* **(B–D)**, *lys-5* **(E)**, and *F53A9.8* **(F)** in wild-type and *scav-5(ok1606)* animals cultured on *E. coli* OP50 or *S. typhimurium* SL1344.

*C. elegans* has an innate immune system and responds to pathogenic bacterial by expression of defense genes (Kim et al., 2002; Ermolaeva and Schumacher, 2014). To test whether loss of function of *scav-5* activates the innate immune response in *C. elegans*, we analyzed the mRNA level of pathogen response genes by quantitative RT-PCR (qRT-PCR). In *C. elegans, clec-7, clec-60*, and *clec-82* encode C-type lectin proteins (Schulenburg et al., 2008), *lys-5* encodes lysozyme (Boehnisch et al., 2011), and *F53A9.8* encode antimicrobial peptides (Zugasti and Ewbank, 2009). Surprisingly, we observed that the expression of pathogen response genes *clec-7, clec-60 clec-82, lys-5*, and *F53A9.8* were lower in *scav-5* mutants compared to wild type when cultured on *E. coli* OP50. And not all but most of the pathogen response genes listed above were downregulated in *scav-5* mutants cultured on *S. typhimurium* SL1344 (**Figures 4A–E**). These indicate that *scav-5* may be a positive regulator for defense genes expression fed on *E. coli* OP50 or *S. typhimurium* SL1344. Intriguingly, we also found that p38 PMK-1 MAPK innate immune response pathway was downregulated in *scav-5* mutants cultured on *E. coli* OP50 or *S. typhimurium* SL1344 by detecting the levels of PMK-1 phosphorylation (**Figure 4F**), which further confirm that *scav-5* is a positive regulator for innate immune response when cultured on *E. coli* OP50 or *S. typhimurium* SL1344.

### Defense Gene Expression Were Upregulated in *scav-5* Mutants Infected by *P. aeruginosa* PA14

**Figure 5:**
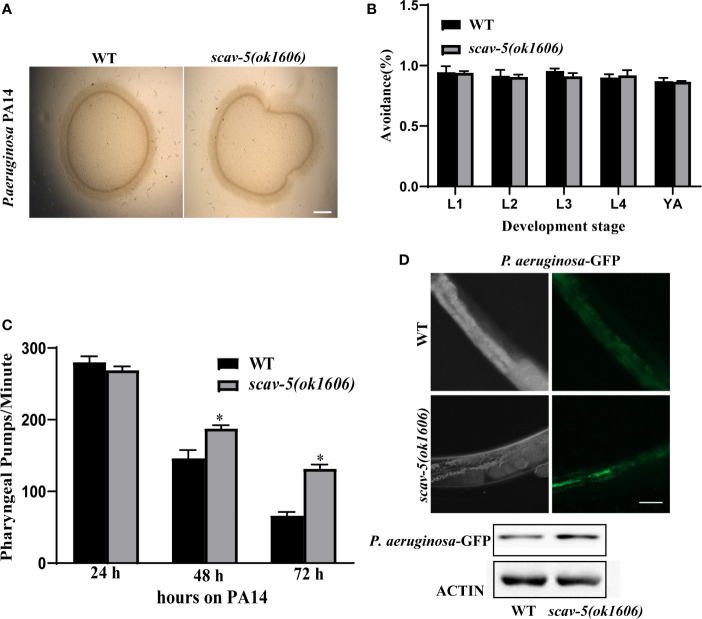
*scav-5* mutants do not display dietary restriction when cultured on *P. aeruginosa* PA14. **(A, B)** Bright field photographs of wild type and *scav-5(ok1606)* mutant avoidance from *P. aeruginosa* PA14 in L4 stage **(A)** and quantification of avoidance rate from L1 stage to young adult **(B)**, Scale bar=100 μm. **(C)** Statistics of pharyngeal pumps per min of wild type and *scav-5(ok1606)* mutants fed with *P. aeruginosa* PA14. **(D)** The DIC and GFP fluorescence image of partial intestine in wild type and *scav-5(ok1606)* mutant cultured on *P. aeruginosa* PA14-GFP 24 h after L4 stage. And immunoblot analysis of protein from young adult animals fed with *P. aeruginosa* PA14 24 h using antibodies that recognize the p-PMK-1 and actin. Scale bar=50 μm.

**Figure 6:**
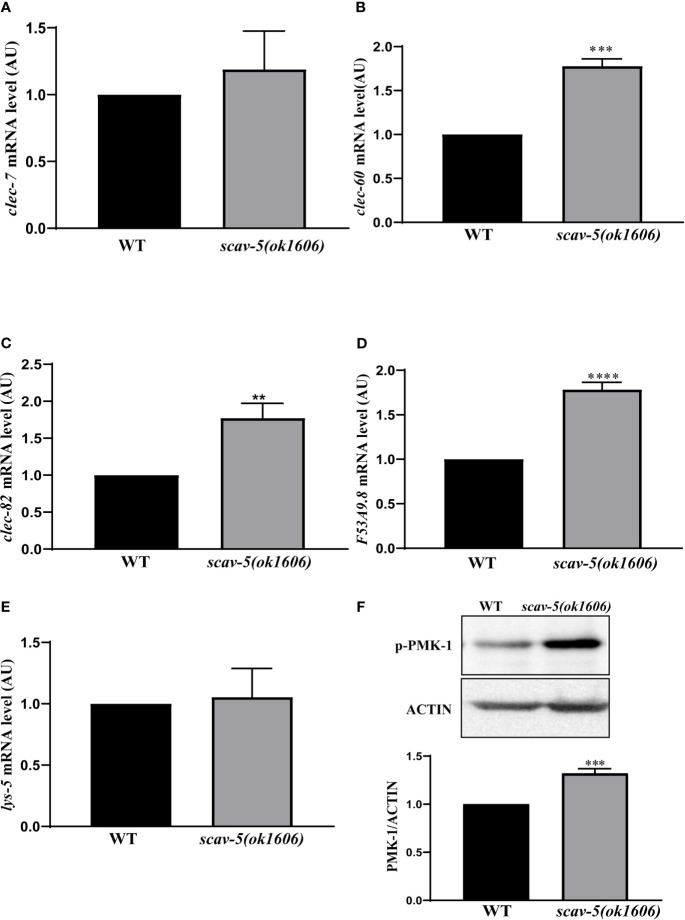
PMK-1 signaling pathways are activated in *scav-5* mutants after *P. aeruginosa* PA14 infection. **(A)** Immunoblot analysis of protein from young adult animals fed with *P. aeruginosa* PA14 24 h using antibodies that recognize the p-PMK-1 and actin. **(B–F)** qRT-PCR analysis of transcription level of pathogenic response genes *clec-7*, *clec-60*, and *clec-82* **(B–D)**, *lys-5* **(E)**, and *F53A9.8* **(F)** in wild-type and *scav-5(ok1606)* animals cultured on *P. aeruginosa* PA14.

To verify the mechanism by which *scav-5* mutants are resistant to *P. aeruginosa* PA14, we first quantified their bacterial lawn avoidance behavior. We found that similar to wild type, *scav-5* mutants animals displayed bacterial avoidance behavior when cultured on *P. aeruginosa* PA14 (**Figures 5A, B**). Next, we analyzed the pharyngeal pumping rate of *scav-5* mutants when fed on *P. aeruginosa* PA14. We observed that compared to WT, *scav-5* mutants displayed significantly higher pharyngeal pumping rate on 48h-72h after L4 stage when cultured on *S. P. aeruginosa* PA14 (**Figure 5C**), indicating that *scav-5* mutants had no dietary restrictions on *P. aeruginosa* PA14. To further confirm that the dietary restriction did not exist in *scav-5* mutants when feeding on *P. aeruginosa* PA14, we detected *P. aeruginosa* PA14 colonization in the intestine of *scav-5* mutants by feeding with *P. aeruginosa* PA14-GFP for 24h after L4 stage. *scav-5* mutants intestine displayed significantly increased fluorescence intensity when cultured on *P. aeruginosa* PA14-GFP (**Figure 5D**). The increased level of *P. aeruginosa* PA14-GFP in *scav-5* mutants was confirmed by Western blotting (**Figure 5D**). These results demonstrate that *scav-5* mutation defend *C. elegans* against *P. aeruginosa* PA14 infection is not by dietary restriction. We then asked whether loss of function of *scav-5* activates the innate immune response in *C. elegans* against *P. aeruginosa* PA14 infection by analyzing the mRNA level of pathogen response genes. We observed that the expression of pathogen response genes *clec-60 clec-82, lys-5*, and *F53A9.8* were higher in *scav-5* mutants when compared to wild type cultured on *P. aeruginosa* PA14 (**Figures 6A–E**). These results reveal that defense genes expression were upregulated in *scav-5* mutants infected by *P. aeruginosa* PA14.

### *scav-5* Mutants Are Resistant to *P. aeruginosa* PA14 Through Activation of PMK-1 p38 MAPK Pathway

**Figure 7:**
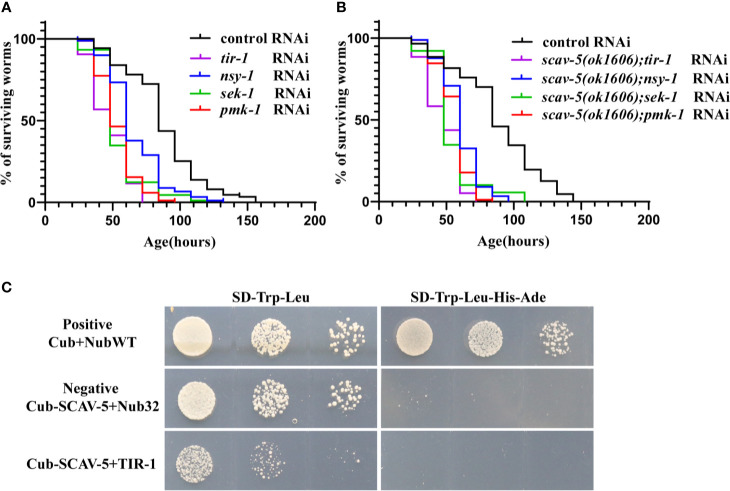
Genetic epistatic analysis *of scav-5* in the PMK-1 pathway defense against *P. aeruginosa* PA14 and test of the interaction SCAC-5 with TIR-1. **(A, B)** Survival curves of wild type **(A)** and *scav-5(ok1606)* mutants **(B)** with *tir-1, nsy-1, sek-1, pmk-1* RNAi cultured on *P. aeruginosa* PA14. **(C)** Analysis of interaction between SCAV-5 and TIR-1 by yeast two hybrid. The yeast solution was diluted by three gradients in each group, which were cultured on SD-Trp-Leu medium (left) and SD -Trp-Leu-His-Ade medium (right).

To explore whether SCAV-5 participates in the innate immune response through the PMK-1 p38 MAPK pathway in *C. elegans* after *P. aeruginosa* PA14 infection, we first measured activated PMK-1 level by immunoblotting. Our results showed that activated PMK-1 in *scav-5* mutants fed on *P. aeruginosa* PA14 was significantly increased compared to wild type control (**Figure 6F**). These data proved that defects can activate PMK-1 p38 MAPK pathways in *C. elegans* infected by *P. aeruginosa* PA14. Next, we test whether *pmk-1* was required for *scav-5* mutants lifespan extension after *P. aeruginosa* PA14 infection. Our results in line with previous studies that loss- and reduction-of-function mutations of p38 MAPK PMK-1 pathway components lead to a reduced lifespan of worms fed on *P. aeruginosa* PA14 (**Figure 7A**) (Xu et al., 2013; Head et al., 2017). Besides, we found *pmk-1* RNAi treatment suppressed the extended lifespan phenotype of *scav-5* mutants when cultured on *P. aeruginosa* PA14 (**Figure 7B**), demonstrating the requirement of *pmk-1* pathway for *scav-5* mutants lifespan extension infected by *P. aeruginosa* PA14. Furthermore, our genetic epistasis analysis suggested *scav-5* function upstream of or in parallel to *tir-1* (**Figure 7B**) (Couillault et al., 2004). Our preliminary analysis of protein structure by the SMART website revealed that SCAV-5 is a membrane protein with two transmembrane domains. We then utilized the split-ubiquitin based membrane yeast two-hybrid (MYTH) system for detecting the interaction of SCAV-5 with TIR-1. However, the result showed that SCAV-5 and TIR-1 did not physically interact (**Figure 7C**). Taken together, our results reveal that *scav-5* mutation protects *C. elegans* against *P. aeruginosa* PA14 by upregulating PMK-1 p38 MAPK pathway.

## Discussion

Mounting evidence shows that many scavenger receptors, including the prototype class B type scavenger receptor CD36, play an important role in innate immunity by serving as the pattern-recognition receptors, in particular against bacterial pathogens. SCAV-1-6 are the six class B scavenger receptors homologs in *C. elegans.* However, it is not clear whether they have effect on host defense against bacterial pathogens. Here, we show that defects in *scav-5* protect worms against pathogenic bacterial *S. typhimurium* SL1344 and *P. aeruginosa* PA14 by different mechanism. *scav-5* mutants are resistant to *S. typhimurium* SL1344 due to dietary restriction. While *scav-5* mutation protects worms against *P. aeruginosa* PA14 by activating the innate immune response through conserved PMK-1 p38 MAPK pathway.

We found that, of the six SCAV proteins homologous, only *scav-5* is involved in innate immune response against pathogenic bacterial. *scav-1, scav-2, scav-4, scav-5* and *scav-6* are expressed in the intestine tissues. It is widely reported that, in response to bacterial infections, *C. elegans* produces an array in intestinal epithelial cells by expressing related antimicrobial genes (JebaMercy et al., 2011). The damage of intestinal epithelium would cause worms more hypersensitive to pathogenic bacteria, which allows live bacteria to enter the intestinal lumen (Kumar et al., 2019). The expression pattern of scav-5 consistent with its function. Indeed, a previous study demonstrates that *scav-1* is necessary for *C. elegans* survival after fungal pathogens infection (Means et al., 2009). It will be interesting to test whether *scav-2, scav-4, scav-5* and *scav-6* participate in defense against other pathogens infection. We observed that SCAV-3 was expressed in all tissues and required for lifespan extension of *C. elegans* when cultured in *E. coli* OP50. Our results in line with the previous study, which also reveals that SCAV-3 is lysosomal membrane protein and is the key regulator of lysosome integrity, motility, and dynamics.

We found that compared to control, the expression of pathogen response genes were downregulated in *scav-5* mutants without infection or cultured on *S. typhimurium* SL1344, either PMK-1 p38 MAPK pathway was activated. These indicate that *scav-5* may be required for the expression of pathogen response genes in worms without infection. This is an important issue for future research. A possible explanation for the downregulation expression of pathogen response genes in *scav-5* mutants fed on SL1344 might be dietary restriction. Bacteria as food for *C. elegans* was ingested by increasing its rate of pharyngeal pumping, which is a process controlled by pharyngeal motor neurons (Kumar et al., 2019). Thus, decreased ingestion *of S. typhimurium* SL1344 in *scav-5* mutants implicate its function may relate to neuron regulation. Further studies will need to determine the underlying mechanism.

We found that the expression of pathogen response genes were upregulated in *scav-5* mutants when cultured on *P. aeruginosa* PA14.This was further confirmed by that *scav-5* acts in PMK-1 p38 MAPK pathway after *P. aeruginosa* PA14 infection, although *scav-5* is required for expression of pathogen response genes under normal condition. As PMK-1 controls basal levels of pathogen response genes on *E. coli* and also induce the upregulation of these genes’ expression upon infection. Our results imply *scav-5* may function in another pathway for controlling the basal level of pathogen response genes. We found that *scav-5* may function upstream of, or in parallel to *tir-1* in PMK-1 innate immune response pathway to protect *C. elegans* against *P. aeruginosa* PA14. However, SCAV-5 and TIR-1 did not physically interact. Further research should be undertaken to investigate how *scav-5* participate in PMK-1 innate immune response pathway.

In summary, our results provide evidence that loss of function of Scavenger receptor SCAV-5 protects *C. elegans* against pathogenic bacteria *S. typhimurium* SL1344 and *P. aeruginosa* PA14 by different mechanisms, establish the link between the SCAV-5 and the innate immune response. To our knowledge, this is the first demonstration of a role for SCAV-5 in host defense against pathogenic bacteria. SCAV-5 is an ortholog of human SRB-I/II, which is implicated in platelet-type bleeding disorder 10 and progressive myoclonus epilepsy 4 (Dibbens et al., 2009; Silverstein and Febbraio, 2009). Thus, our research provides important clues for further dissecting the mechanism by which SRB-I/II regulates innate immune responses.

## Data Availability Statement

The raw data supporting the conclusions of this article will be made available by the authors, without undue reservation.

## Author Contributions

HX and QZ conceived the study. AL did most of the experiments. HJ did the manuscript revision and experimental repeats. LY and YW contributed to materials. HX, QZ, and AL wrote the manuscript with feedback from all authors. All authors contributed to the article and approved the submitted version.

## Funding

This work was partially supported by the National Natural Science Foundation of China (Grant No. 31671439 to HX), National Natural Science Foundation of China Youth Program (Grant No. 31801164 to QZ), Natural Science Foundation of Shaanxi Province, China (Grant No. 2020JM-271 to HX), the program of Innovative Research Team for the Central Universities (Grant No. GK202001004 to HX), the Fundamental Research Key Project Funds for the Central Universities (Grant No. GK202007009 to HX).

## Conflict of Interest

The authors declare that the research was conducted in the absence of any commercial or financial relationships that could be construed as a potential conflict of interest.

## Publisher’s Note

All claims expressed in this article are solely those of the authors and do not necessarily represent those of their affiliated organizations, or those of the publisher, the editors and the reviewers. Any product that may be evaluated in this article, or claim that may be made by its manufacturer, is not guaranteed or endorsed by the publisher.
