# Supplementary material for: Loss of Function of Scavenger Receptor SCAV-5 Protects C. elegans Against Pathogenic Bacteria
Source: Front Cell Infect Microbiol. 2021 Aug 3;11:593745. doi: 10.3389/fcimb.2021.593745 (PMC8370389; doi:10.3389/fcimb.2021.593745)
Supplement: Supplementary file 1 [file Table_1.docx]

**Supplemental Information：**

**Loss of function of Scavenger receptor SCAV-5 protects *C. elegans* against pathogenic bacteria**

Aixiao Luo^1*^, Huiru Jing^1*^, Lei Yuan^1^, Yanzhe Wang^1^, Hui Xiao^1#^, Qian Zheng^1#^


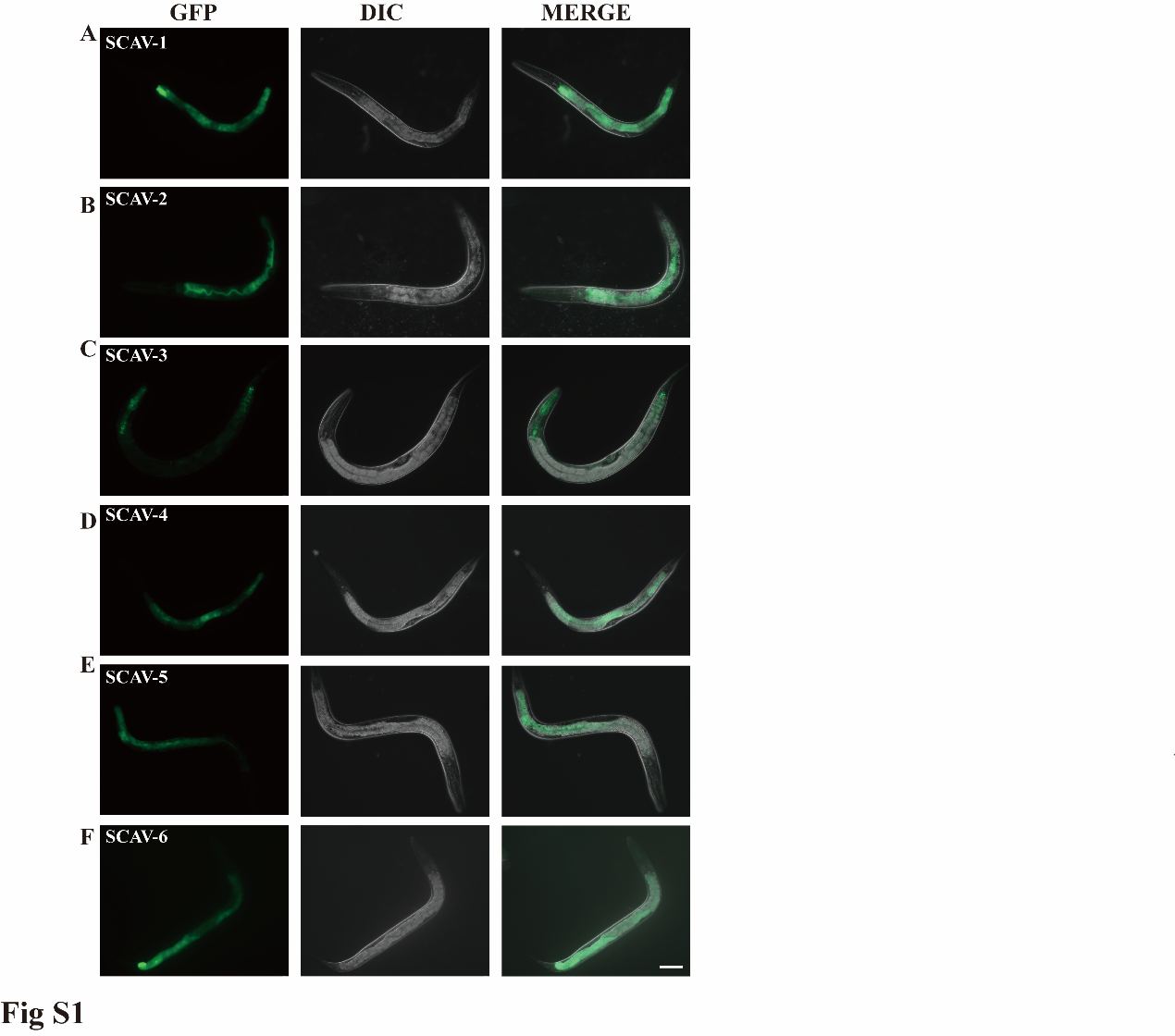


**Figure S1** The expression pattern of scavenger receptors in L4 *C.elegans*.

**(A-F)** Tissue profiling of scavenger receptor SCAV-1-6. DIC images of L4 stage live transgenic animals(left). Green fluorescence images show tissue localization of scavenger receptor SCAV-1-6(middle). Merge images(right). Scale bar=50 μm.


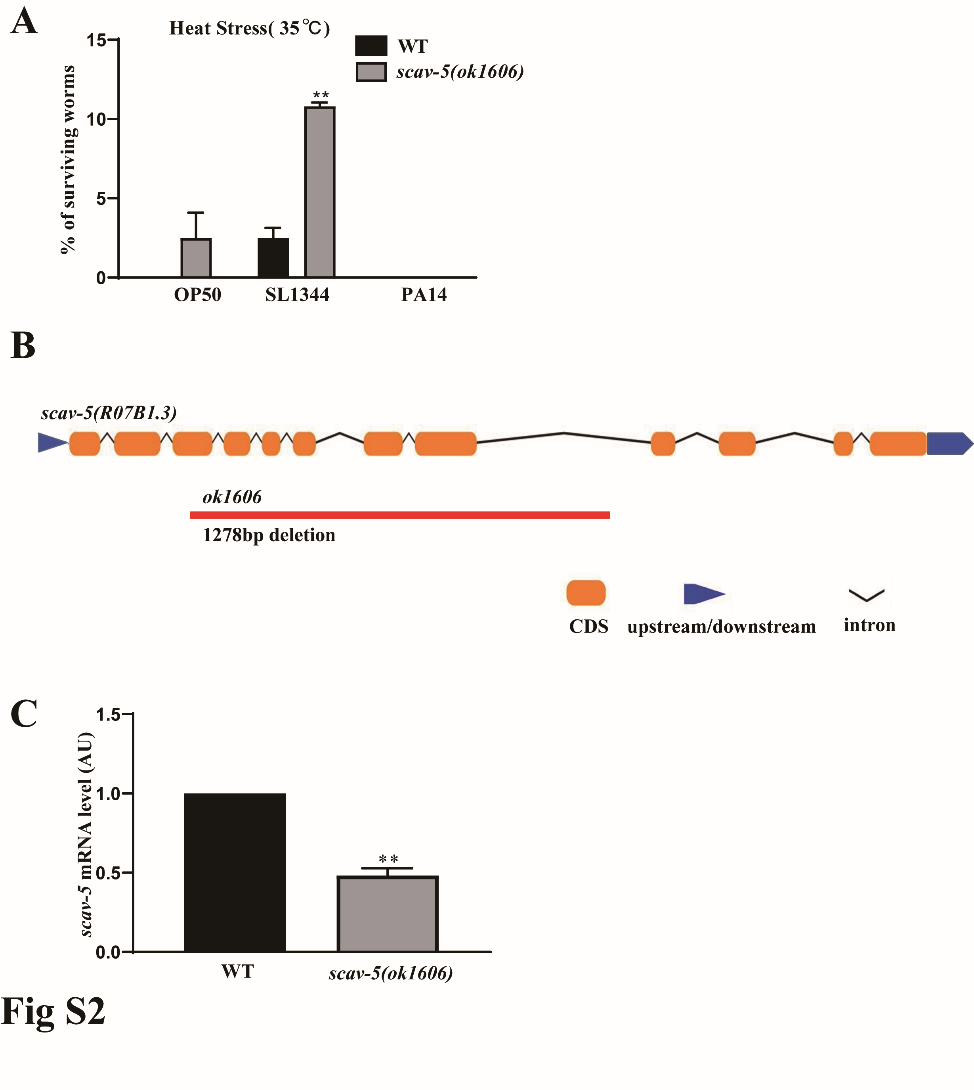


**Figure S2** *scav-5(ok1606)* is likely to be a strong loss of function or null allele.

**(A)** Survival rate of *scav-5* mutants deal with heat resistance 12 h at 35℃ when feeding on *E. coli* OP50, *S. typhimurium* SL1344 48 h and *P*. *aeruginosa* PA14 24 h after L4 stage respectively.

**(B)** Scheme of the deleted allele of *scav-5(ok1606).*

**(C)** The lack of detectable *scav-5* mRNA in *scav-5(ok1606)* by QPCR. Statistical significance was determined using t-tests. **, P < 0.01


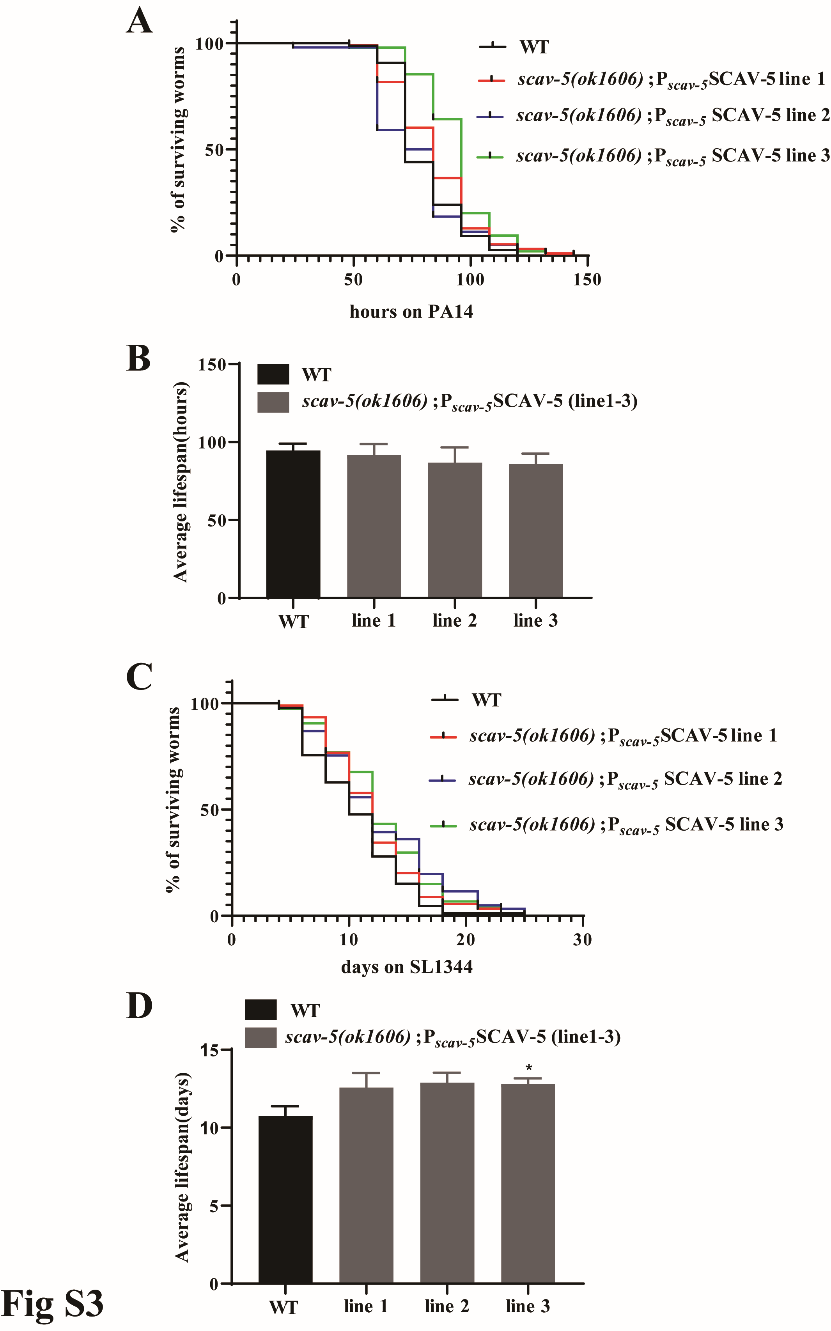


**Figure S3** The lifespan analysis of *scav-5(ok1606)*;P*_scav-5_*SCAV-5 fed on *S.* *typhimurium* SL1344 and *P. aeruginosa* PA14.

**(A and C)** Survival curve of *scav-5(ok1606)*;P*_scav-5_*SCAV-5 by *S. typhimurium* SL1344 (A) and *P. aeruginosa* PA14 (C) infection.

**(B and D)** Average lifespan of *scav-5(ok1606)*;P*_scav-5_*SCAV-5 animals by *S. typhimurium* SL1344 (B) and *P. aeruginosa* PA14 (D) infection.*, P < 0.05
